# Comparative Analysis of GF-1 and HJ-1 Data to Derive the Optimal Scale for Monitoring Heavy Metal Stress in Rice

**DOI:** 10.3390/ijerph15030461

**Published:** 2018-03-06

**Authors:** Dongmin Wang, Xiangnan Liu

**Affiliations:** School of Information Engineering, China University of Geosciences, Beijing 100083, China; wdm@cugb.edu.cn

**Keywords:** remote sensing, optimal scale, heavy metal stress, WOFOST model

## Abstract

Remote sensing can actively monitor heavy metal contamination in crops, but with the increase of satellite sensors, the optimal scale for monitoring heavy metal stress in rice is still unknown. This study focused on identifying the optimal scale by comparing the ability to detect heavy metal stress in rice at various spatial scales. The 2 m, 8 m, and 16 m resolution GF-1 (China) data and the 30 m resolution HJ-1 (China) data were used to invert leaf area index (LAI). The LAI was the input parameter of the World Food Studies (WOFOST) model, and we obtained the dry weight of storage organs (WSO) and dry weight of roots (WRT) through the assimilation method; then, the mass ratio of rice storage organs and roots (SORMR) was calculated. Through the comparative analysis of SORMR at each spatial scale of data, we determined the optimal scale to monitor heavy metal stress in rice. The following conclusions were drawn: (1) SORMR could accurately and effectively monitor heavy metal stress; (2) the 8 m and 16 m images from GF-1 were suitable for monitoring heavy metal stress in rice; (3) 16 m was considered the optimal scale to assess heavy metal stress in rice.

## 1. Introduction

Heavy metals in soils have become major environmental pollutants that can affect crops throughout the world [1,2]. Therefore, rapid and effective monitoring of heavy metal pollution in rice has important and practical significance. Traditional methods for monitoring heavy metal stress involve collecting rice samples and conducting laboratory analyses [3,4]. Although the traditional methods have high precision and the technology is mature, it is difficult to extrapolate the results to a large scale. Remote sensing can remotely detect target objects and dynamically monitor a large area in real-time, which provides a new method for rapid and large-scale monitoring of environmental pollution [5,6]. Several studies have explored the physiological and ecological parameters of rice under heavy metal stress and the corresponding spectral response characteristics from different perspectives [7,8,9,10]. The studies show that under heavy metal stress, the spectral reflectance increased with the increase of pollution in the red band, while in the near infrared, the reflectance of spectral reflectance decreased significantly. The characteristics of remote sensing information under rice pollution stress have been revealed, and studies indicate that the use of remote sensing technology to monitor heavy metal pollution in rice is principally and technologically feasible [11,12]. However, for many remote sensing data products, the problem of how to select the appropriate data to research heavy metal stress in rice, that is, the scale problem, needs to be solved.

Scale has different meanings in different fields. It is a difficult problem in studying remote sensing and is the basis of remote sensing information analysis and application [13,14]. This problem has provoked great concern internationally and is a key factor in the analyses of remote sensing applications [15,16]. Therefore, the exploration of scale has very important scientific significance. The studies related to scale mainly include scale transformation, scale effect, multi-scale modeling, scale analysis, and so on [17]. Scale also changes with the research objects. Moreover, the choice of scale directly affects the results of remote sensing analyses.

Each feature has characteristics that are only shown at a specific scale. When the same object is observed at different scales, the results may be inconsistent, and different attributes will appear [18]. For observational results, if the surface is homogeneous, a point can represent the surface, so there is no scale effect; if the surface is heterogeneous, there is a significant scale effect. This is the meaning of spatial heterogeneity [19,20]. Due to spatial heterogeneity, it is necessary to adopt an appropriate scale to achieve the best observation of different research objects [21,22]. It may be ineffective to select a spatial resolution that is easy to obtain based on an existing remote sensing image [13,23]. Therefore, it is necessary to research the optimal scale of an object, which is a difficult problem to solve in the field of remote sensing. Lam et al. [24] defined four kinds of space-related scale and one is the resolution: the higher the resolution the smaller the scale. Therefore, the optimal scale is equal to the optimal resolution. This study mainly focuses on spatial scale, and the optimal scale in this study refers to the optimal spatial resolution.

There have been many studies on the optimal scale in remote sensing, including for environmental monitoring [25,26] and assessing physiological parameters of vegetation [27,28]. At present, the optimal scale selection method mainly includes local variance [16,18], variation function [21,29], statistical analysis [30,31], etc. Certainly, there are still some limitations in the current methods of optimal scale selection. Han et al. [14] suggested that the actual spatial distribution of the observation volume should be considered in the practical application of remote sensing. Therefore, when choosing the remote sensing data, the optimal scale domain should be judged according to the actual study area. So according to the situation in the study area, it is better to select the medium and high resolution remote sensing images.

Presently, many medium-resolution and high-resolution satellite sensors are orbiting the Earth, including the Environment and Disaster Monitoring and Forecasting Satellite (HJ-1) series, the Chinese high-resolution Earth observation system Satellite 1 (GF-1), the Resources satellite three (ZY-3) and so on. As representatives of the Chinese high-resolution satellites, GF-1 and HJ-1 play important roles in the earth observation system, especially in monitoring crop growth for agriculture [32,33,34]. However, it is necessary to exclude the differences in the orbital altitudes of the sensors, the spatial and spectral resolutions, the spectral responses of the sensors, and the wavelength band limits when using the images from both satellite sensors [35,36]. He [27] noted that the spectral response function and radiation calibration have small effects on the band reflectance values, vegetation indexes and LAI results, and these can be ignored in practical applications, but the spatial resolution was found to have a significant influence on the LAI results between the different sensors and must be taken into consideration. Some studies mainly used the GF-1 WFV and HJ-1 CCD sensor data for monitoring the environment [25,26,27]. In general, few studies have used the combination of the GF-1 wide field of view (WFV) camera, the full color multispectral (PMS) camera and the HJ-1 charge-coupled device (CCD) data to monitor heavy metal stress and determine the optimal scale for monitoring.

The objective of this paper was to obtain the optimal scale to assess heavy metal stress in rice. Therefore, this paper includes (1) an inversion of LAI using 2 m, 8 m, and 16 m resolution GF-1 data and 30 m resolution HJ-1 data; (2) an assimilation method used to extract the sensitive characteristics of heavy metal stress, as data assimilation provides a method to incorporate information from both models and observations [37,38,39]; and (3) the selection of an optimal spatial scale for remote sensing monitoring of heavy metal stress in rice.

## 2. Study Area and Data

### 2.1. Study Area

The selected test sites are situated in Zhuzhou, which is located in the east of Hunan Province, China. Zhuzhou is an industrial base with serious heavy metal pollution, especially near the Xiangjiang River basin. Zhuzhou has a subtropical humid monsoon climate with an average annual temperature of 16 °C to 18 °C and an average annual precipitation of 1400–1700 mm. It has long frostless periods with an average of more than 286 days per year. It is suitable for rice growth, and it is a famous grain base in China.

**Figure 1:**
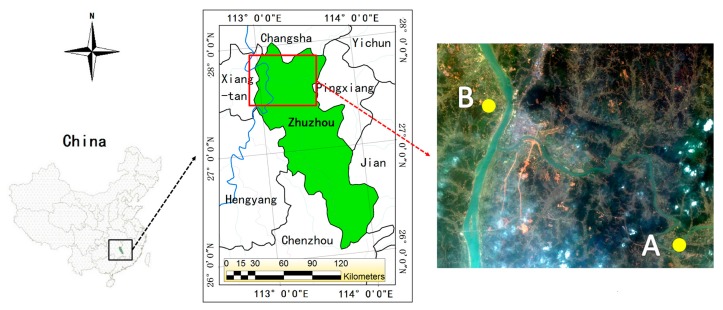
Location of the study area and the remote sensing image used in this study.

Two relatively large areas were selected as the study area (Figure 1). Area A (27°37′ N, 113°15′ E) is far away from the Xiangjiang River but is near the Lushui River, and area B (27°43′ N, 113°7′ E) is close to the Xiangjiang River. The two study areas are nearly rectangular, with area A approximately 280 × 480 m^2^ and area B approximately 460 × 660 m^2^. According to the measured data, the heavy metal content in area B is much higher than the background value. Similarly, the heavy metal content of the rice in area B is also high (Table 1), and area B is heavily polluted compared with area A. Therefore, the background value is used as the evaluation criterion, and area A and area B are rated to have low pollution and high pollution levels, respectively. As the climate, topography, and field management techniques are similar in the two areas, the other types of stress on the growth of rice are relatively small and can be ignored.

### 2.2. Remote Sensing Data

This study used four different scales of remote sensing image data from two different sensors, namely, 2 m, 8 m, and 16 m GF-1 images and a 30 m HJ-1 image. GF-1 was the first satellite in China’s high-resolution earth observation system and was launched in 2013. The satellite has two, 2 m panchromatic/8 m multispectral cameras and four 16 m multispectral cameras. HJ-1 includes two optical satellites HJ-1A/B and one radar satellite HJ-1C, that can dynamically monitor the environment and disasters over a large scale, in all weather conditions and at all times of the day. The design principles of the two CCD cameras mounted on HJ-1A and HJ-1B are identical. The attributes of the images that were used in this study are shown in Table 2.

The study areas contained relatively small areas of rice paddy fields, so the use of high-resolution remote sensing images could ensure the consistency of rice varieties. Three remote sensing images were selected for each spatial resolution. Three important rice phenological stages were monitored: the tillering stage (mid-June), the jointing–booting stage (mid-July) and the ripening stage (late August). GF-1 images taken on 15 June, 18 July and 28 August in 2014 were selected. HJ-1 images taken on 13 June, 22 July and 29 August in 2014 were selected. Four images at almost the same time were selected to avoid time errors. The two meter GF-1 images are a fusion of the two meter full-color images and the eight meter multispectral images. All images were subjected to data preprocessing, including radiation correction, atmospheric correction and geometric correction. The atmospheric correction was done using the fast line-of-sight atmospheric analysis of spectral hypercubes (FLAASH) model. The geometric correction of the GF-1 image was conducted using orthorectification. Namely, rational polynomial coefficient (RPC) ortho-rectification with a digital elevation model (DEM) file was used. All operations were performed in ENVI 5.3.

### 2.3. Measured Data

The measured field data and the meteorological data of the study area are also required. All data were obtained between June and September 2014.

The measured data included the relevant soil and rice parameters, and the acquisition time of the parameters was consistent with the remote sensing image acquisition time. A total of 30 evenly distributed sampling points were selected in each study area using a global positioning system (GPS). Soil and rice samples were collected at each sampling point, and the depth of soil samples is about 10 cm. The rice was dried at room temperature, and the weight of the roots, stems, leaves and storage organs were obtained separately. All parts of the dried rice were sealed and sent to the Chinese Academy of Agricultural Sciences for heavy metal content analysis along with the soil.

### 2.4. Meteorological Data

Meteorological data were important input parameters for the crop growth model (WOrld FOod STudies (WOFOST)). The meteorological data include atmospheric temperature (T) and solar radiation. The meteorological data were obtained from the China Meteorological Data Sharing Service System.

## 3. Methods

It is well known that heavy metals can reduce plant growth rates and biomass yields [40]. First of all, this study accurately defines the characteristics of the target, that is, the main characteristics of rice under heavy metal stress. Many studies have shown that, compared with other parts, the highest concentration of heavy metals is in the roots [41,42,43]. However, the root as the only characteristic indicator would overestimate heavy metal stress [44]. The edible parts of rice are storage organs, and the storage organs are developed after the rice is transplanted. Therefore, to study the heavy metal stress throughout the growing season, the rice storage organs and underground biomass data were selected as the indicators of heavy metal stress, but these data were difficult to obtain directly. So we used the improved WOFOST (crop growth) model to simulate the dry weight of roots (WRT) and the dry weight of storage organs (WSO). Compared with the aboveground biomass, rice roots are more sensitive to heavy metal reactions. However, the weight of the roots was not chosen as the sole indicator of heavy metal stress because low concentrations of heavy metals can promote rice growth [45,46,47]. Therefore, we used the WSO/WRT as the index of heavy metal stress in this paper to reduce the error caused by low concentrations of Pb and other heavy metals that can promote rice growth.

### 3.1. Computing LAI from GF-1 and HJ-1 Data

Obtaining the LAI from remote sensing images is an important step in the data assimilation process in this article because LAI is an important input parameter for the WOFOST model. The correct inversion of LAI is critical to monitor crop growth. Many studies have shown that the use of the normalized difference vegetation index (NDVI) inversion of LAI is undesirable because the sensitivity of NDVI to LAI decreases and saturation occurs when the value of LAI is higher than 2 or 3 [48,49]. To avoid this problem, many researchers have conducted blue NDVI (BNDVI), green NDVI (GNDVI), blue-green NDVI (GBNDVI), red-blue NDVI (RBNDVI), green-red NDVI (GRNDVI), and green-red-blue (PNDVI) tests, and these tests found that GBNDVI and GNDVI had high accuracy in retrieving LAI [48]. It also has been found that, for HJ-1 images, the use of the inversion of LAI from GNDVI is better than that from GBNDVI [50]. In addition, we did not use the blue band, because the blue band is strongly influenced by atmospheric scattering. To control the variables, whether it was a GF-1 or HJ-1 image, this paper used GNDVI to invert the LAI. The GNDVI and LAI formulas [51] are as follows:

(1) $$\mathrm{GNDVI}=\frac{\rho_{\mathrm{NIR}}-\rho_{G}}{\rho_{\mathrm{NIR}}+\rho_{G}},$$

(2) $$\mathrm{LAI}=0.508e^{3.384\mathrm{GNDVI}},$$

where $\rho_{\mathrm{NIR}}$ and $\rho_{G}$ are the reflectance values of the near-infrared and green bands, respectively. In this paper, we calculated the four different resolutions of GNDVI and their corresponding LAI values.

### 3.2. Extraction of WRT and WSO

The crop growth model (WOFOST) [52] can quantitatively simulate some of the parameters in the crop growth process according to the input parameters. The WOFOST model has three simulated crop growth levels, namely, potential productivity levels, water stress levels and nutrient limit levels, and the potential productivity level is the main factor that restricts the growth of the crop by solar radiation and temperature. The WOFOST model will be used to simulate the WRT and WSO required in this paper. To accurately simulate crop growth, the improved WOFOST [44] model was used. In the improved WOFOST model, two stress factors were added at the potential growth level, which were the total daily gross assimilation of CO_2_ $f_{\mathrm{DTGA}}$ (Equation (3)) and the carbohydrate-to-dry matter conversion coefficient $f_{\mathrm{CVF}}$ (Equation (4)). We combined the two stress factors into f (Equation (5)); the relevant equations are as follows:

(3) $$f_{\mathrm{DTGA}}\left(D\right)=f_{\mathrm{DTGA}}\times \mathrm{DTGA}\left(D\right){\;\;\;f}_{\mathrm{DTGA}}\in \left(0.7,1\right),$$

(4) $$f_{\mathrm{CVF}}\left(D\right)=f_{\mathrm{CVF}}\times \mathrm{CVF}\left(D\right){\;\;\;f}_{\mathrm{CVF}}\in \left(0.7,1\right),$$

(5) $$f=f_{\mathrm{DTGA}}\times f_{\mathrm{CVF}},$$

where D is the day of the year, $f_{\mathrm{DTGA}}\left(D\right)$ and $f_{\mathrm{CVF}}\left(D\right)$ are the total daily gross assimilation of CO_2_ and the carbohydrate-to-dry matter conversion coefficient under stressed growth conditions on D, respectively; DTGA(D) and CVF(D) are the corresponding parameters under the potential growth level.

To accurately simulate the potential growth of rice in the study area, the parameters in the WOFOST model such as leaf area, dry matter distribution coefficient, net photosynthetic rate and the meteorological conditions related to rice variety were consistent with the measured data. The LAI values from four different remote sensing images with different spatial resolutions on three similar dates were used as the input parameters for the improved WOFOST model. According to the input LAI, the WRT and WSO that were required in this study were simulated in the improved WOFOST model.

**Figure 2:**
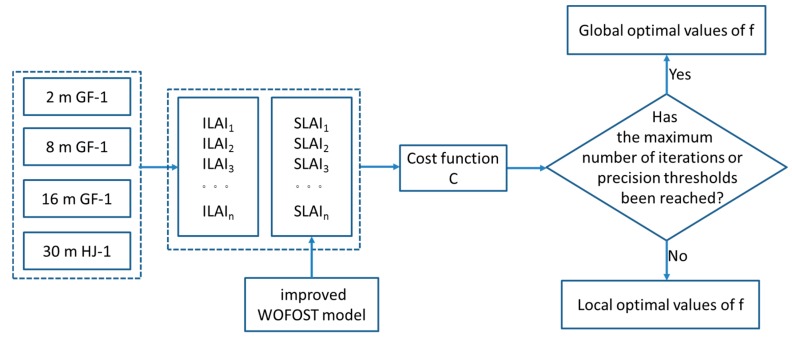
The particle swarm optimization (PSO) assimilation algorithm.

In the improved WOFOST model, the particle swarm optimization (PSO) assimilation algorithm [53] was used to determine the optimal value of the stress factor. The PSO assimilation algorithm minimizes the values of the cost function C by continually adjusting and optimizing the initial parameters in the WOFOST model. The stress factor is constantly adjusted as far as possible to simulate the LAI and keep the remote sensing image LAI inversion consistent. The expression of cost function C is shown as follows:

(6) $$C=\sqrt{\frac{1}{N}\sum_{i=1}^{N}\sum_{j=1}^{M}{\left({\mathrm{LAI}}_{I}-{\mathrm{LAI}}_{S}\right)}^{2}},$$

where N is the total number of available remote sensing images across the entire rice growth period, which was equal to 3. M represents the total number of LAI images at different resolutions, and the value was 4 in this study (2 m, 8 m, 16 m and 30 m). ${\mathrm{LAI}}_{I}$ is the value of LAI inverted from the j m image, and ${\mathrm{LAI}}_{S}$ represents the value of the LAI simulated by the WOFOST model at the corresponding spatial resolution. Figure 2 shows the PSO assimilation algorithm, and the assimilation process ended when the global optimal values of f were obtained. Detailed descriptions of the improved WOFOST model and the PSO assimilation algorithm are provided in the references [54].

### 3.3. Determination of the Heavy Metal Stress Condition Indicators

The temporal and spatial simulation of WRT and WSO are of great significance to the production and management of agricultural grain. Based on the meteorological data and crop phenology information of the study area, the improved WOFOST model [44] combined with two stress factors was calibrated to simulate the WRT and WSO. The expected results were as follows; the simulated WRT value is in the range of 3–350 g/$m^{2}$ [51], and the simulated WSO value is in the range of 0–1000 g/$m^{2}$ [55]. To better compare the qualities of the roots and the storage organs, this paper designed a new indicator, SORMR (the mass ratio of storage organs to roots). In the input parameters of the improved WOFOST model, we set the transplant date to the 154th day, so the value of WRT was assigned on the 154th day, and the WSO has a value of approximately the 200th day. To better compare the WRT and WSO, we divided the weight of the storage organ molecules by the weight of the roots. The formula was as follows:

(7) $$\mathrm{SORMR}\;=\frac{\mathrm{WSO}}{\mathrm{WRT}}.$$

In order to quantify the heavy metal stress in rice under different spatial scales, we conducted numerical statistical analysis and ratio analysis of SORMR. The minimum, maximum, average and standard deviation of SORMR values at each scale in area A and area B were calculated and analyzed. The purpose of ratio analysis is to visualize the differences in heavy metal stress in area A and area B. Finally, the results of the analysis at different spatial resolutions in the two different study areas were used to judge the heavy metal stress.

## 4. Results

### 4.1. WRT Analysis

**Figure 3:**
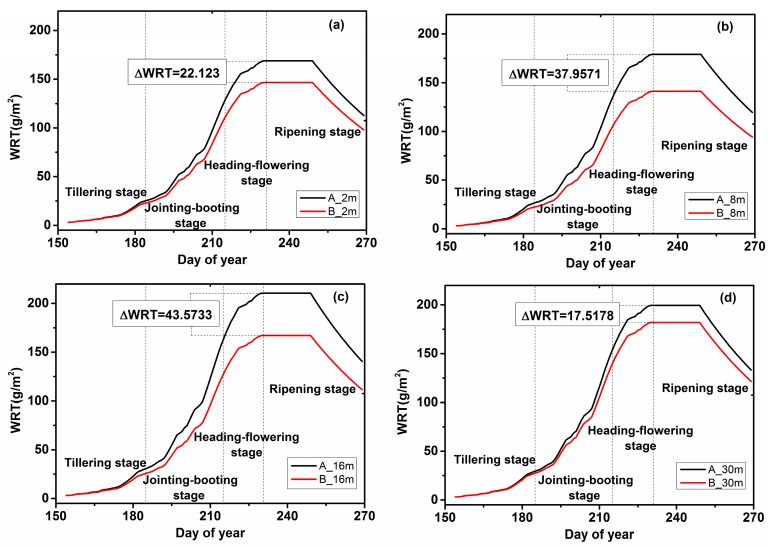
The effects of different spatial resolutions (2 m, 8 m, 16 m and 30 m) on the estimation of dry weight of roots (WRT) values in the two study areas; (**a**) 2 m; (**b**) 8 m; (**c**) 16 m; (**d**) 30 m.

Based on the meteorological data and the crop phenology information from the study area, the improved WOFOST was calibrated, and the two stress factors were used to simulate the assimilation of WRT. Through the improved WOFOST model, we simulated the weight of the rice roots in study areas A and B at the four spatial resolutions. Figure 3 shows the curves of the average simulated WRT values for the rice in each study area at different spatial resolution. All simulated WRT values exhibited similar growth trends at different spatial resolutions, namely, growth, stability, and then decline. In the early stages of rice growth, the simulated WRT values were low, and there were almost no differences between the different spatial resolutions in the different regions. During the jointing–booting stage, the growth of rice increased rapidly, and the WRT growth rate reached the maximum. At this time, the WRT value of area A was higher than that of area B, which indicated that the rice roots were under stress. Then, during the heading–flowering stage, the WRT values were essentially constant. After entering the ripening stage, the roots aged gradually, and the WRT values decreased.

As shown in Table 3, compared with those in area B, the stress factors in area A were closer to 1 and approached the potential productivity level. This indicated that heavy metal stress in area B is more serious than in area A. As shown in Figure 3, the WRT values in areas A and B began to show significant differences at each spatial resolution when the rice entered the jointing–booting stage. It could be seen from the 8 m and 16 m images that the WRT values of area A and area B began to appear different in the late tillering stage. When the rice entered the ripening stage, the difference in WRT between areas A and B was the largest, and the WRT values at the four spatial resolutions went in the order of 16 m > 8 m > 2 m > 30 m.

This observation showed that the simulated WRT values based on remote sensing data and the WOFOST model assimilation could be used to assess heavy metal stress levels. If WRT values are used to evaluate heavy metal stress, the 16 m image was the best remote sensing image. However, using WRT values alone was far from enough, because WRT values could overestimate the impact of heavy metal stress.

### 4.2. WSO Analysis

The WSO analysis was similar to the WRT analysis, and the improved WOFOST model was also calibrated according to the meteorological data and crop phenology information of the study area, and the two stress factors were used to simulate the assimilation of WSO. Figure 3 shows the curves of the average simulated WSO values for rice in each study area at different spatial resolutions. All simulated WSO values also exhibited similar growth trends at different spatial resolutions. Unlike WRT, the WSO grew during the tillering stage, and almost did not change during the jointing–booting stage. Then, during the heading–flowering stage, WSO grew slowly, and there were almost no differences between the different spatial resolutions in the different regions. After entering the ripening stage, the WSO grew faster and continued to grow, reaching the maximum.

**Figure 4:**
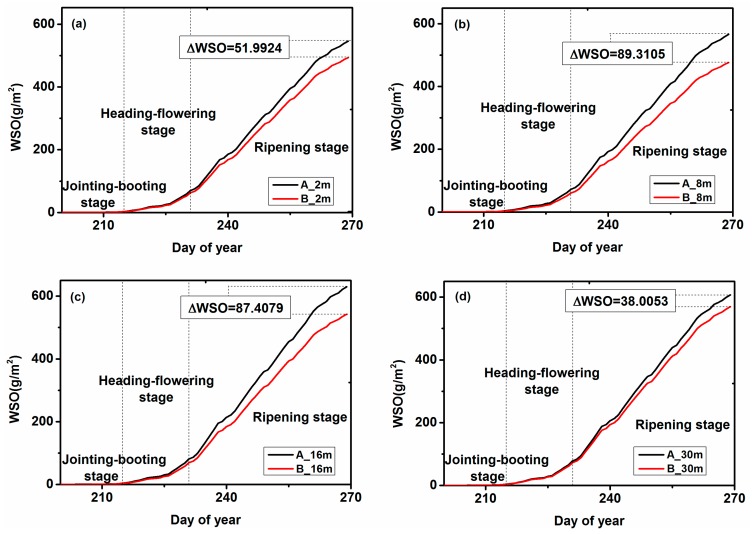
The effects of different spatial resolutions (2 m, 8 m, 16 m and 30 m) on the estimation of dry weight of storage organs (WSO) in the two study areas; (**a**) 2 m; (**b**) 8 m; (**c**) 16 m; (**d**) 30 m.

As shown in Figure 4, the WSO values of area A and area B were different after the rice entered the ripening stage, and the WSO value of area A was more than that of area B. This leads to the conclusion that heavy metals affect the growth of rice storage organs and that result can be obtained at each spatial resolution. When the rice enters the ripening stage, the growth rates of the rice storage organs became faster, and the growth rates under the different heavy metal stress levels are different. That is, if we use WSO as an indicator of heavy metal stress, we should select the ripening stage of rice.

At the four different spatial resolutions, there was a difference in the WSO between area A and area B from the ripening stage. The differences between the maximum values were also different, and the difference between the 8 m and 16 m images was the largest. This indicated that if WSO was used to judge the heavy metal stress index, 8 m and 16 m were the optimal spatial resolutions.

The above results showed that the simulated WSO values based on the remote sensing data and the WOFOST model could be used to evaluate the heavy metal stress levels, and 8 m and 16 m were the optimal scales. However, the use of WSO was also not enough by itself.

### 4.3. SORMR Analysis

**Figure 5:**
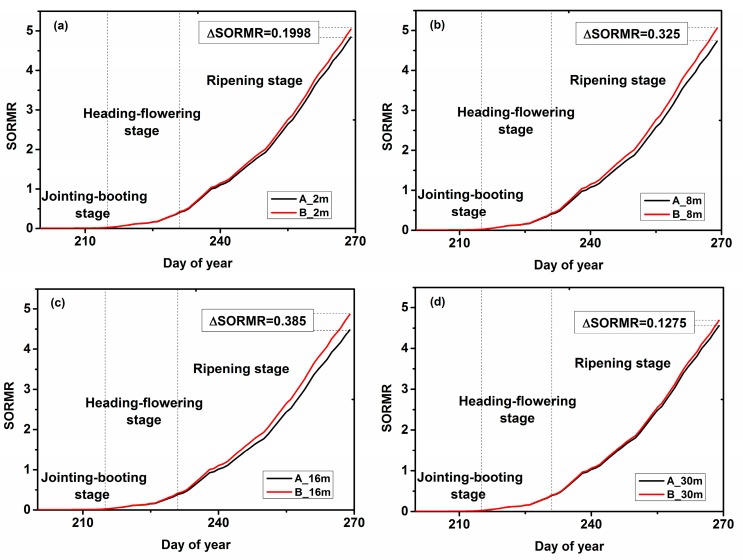
The effects of different spatial resolutions (2 m, 8 m, 16 m and 30 m) on the mass ratio of storage organ and root (SORMR) estimation of the two study areas; (**a**) 2 m; (**b**) 8 m; (**c**) 16 m; (**d**) 30 m.

Similarly, since the dry weight value of the storage organ was measured around the jointing–booting stage, SORMR also began at the jointing–booting stage. Therefore, we decided that the fitted curve would begin at 200. According to the graphs of the SORMR values calculated from the simulated WRT and WSO values (Figure 5), although the spatial resolutions were different, the trends of the curves were consistent. The values of SORMR are between 0–5.5 and present an increasing trend. When the rice entered the ripening stage, the SORMR values of area A and area B were different, and the SORMR value of area A was smaller than that of area B. It was concluded that SORMR could be used to determine heavy metal stress. In addition, we noticed that the 8 m and 16 m images produced quite different results.

**Figure 6:**
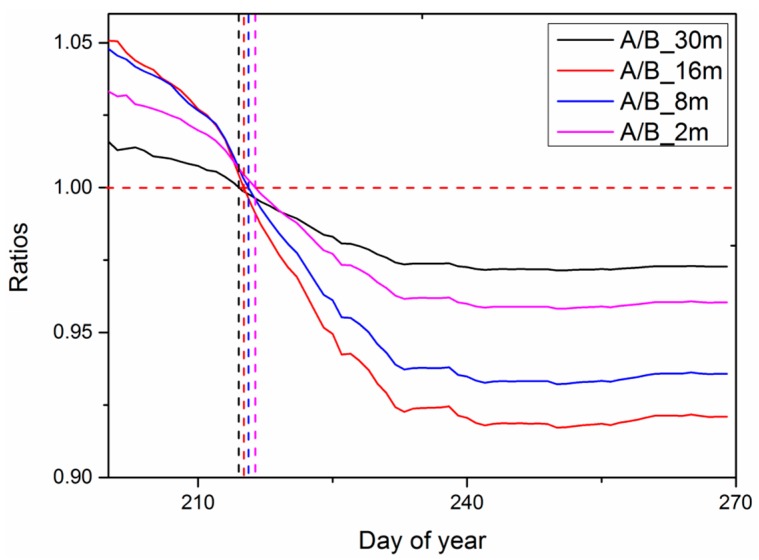
The ratios of SORMR for the two study areas.

To identify the characteristic scale most appropriate to detect heavy metals, the ratio of SORMR in area A and area B was analyzed. As shown in Figure 6, when the ratio was close to 1, the SORMR of the two regions was similar. If the ratio was less than 1 or greater than 1, the difference between the two regions was larger. The lowest ratio was observed at the spatial resolution of 16 m, when the ratio decreased to 0.92 during the ripening stage. The results from the 8 m image were slightly worse than those from the 16 m image.

**Figure 7:**
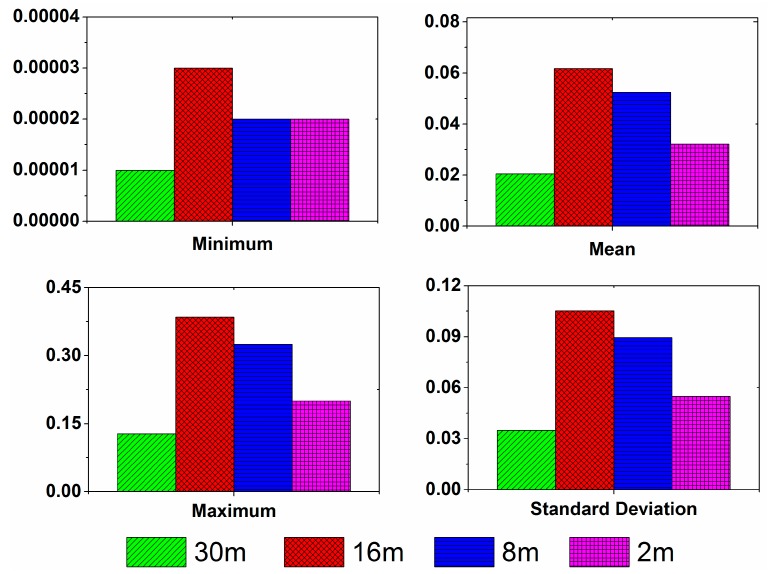
The absolute value of the differences between areas A and B.

The minimum, mean, maximum, and standard deviation values of the SORMR values at different spatial resolutions are shown at Table 4, where the minimum is zero, so we chose the non-zero minimum. Then, we analyzed the results of the statistical analysis, and the absolute value of the difference between areas A and B is obtained. The result is shown in Figure 7. It can be seen from the figure that the differences between the mean values, the maximum values and the standard deviations of SORMR are largest at the 16 m spatial resolution.

## 5. Discussion

Heavy metal pollution is one of the main problems of food security, which is closely related to human health [56]; so effective monitoring of heavy metal pollution is very important. The increase in the number of satellite sensors allows us to diversify remote sensing research and use a wider range of spatial resolutions. However, the results of heavy metal stress differ when using data at different spatial resolutions. In this study, the optimal scale for heavy metal stress detection in rice at a high resolution was tentatively identified.

**Figure 8:**
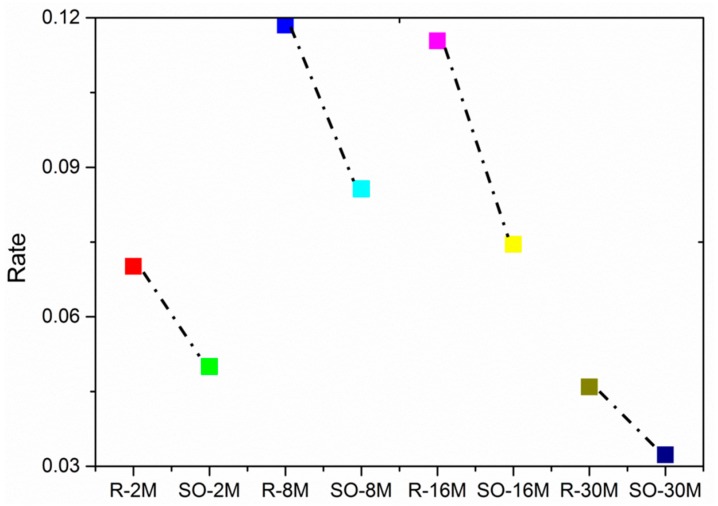
The value of $\frac{\left|{\mathrm{WRT}}_{A}-{\mathrm{WRT}}_{B}\right|}{{\mathrm{WRT}}_{A}+{\mathrm{WRT}}_{B}}$ and $\frac{\left|{\mathrm{WSO}}_{A}-{\mathrm{WSO}}_{B}\right|}{{\mathrm{WSO}}_{A}+{\mathrm{WSO}}_{B}}$ at different scales; R-2M, R-8M, R-16M, R-30M respectively represents the ratio of WRT at 2M, 8M, 16M, 30M scale; SO-2M, SO-8M, SO-16M, SO-30M represents the ratio of WSO at 2M, 8M, 16M, 30M scale.

Few studies have focused on the WRT as the representative characteristic for heavy metal stress [43]. However, in the course of this study, we could not rule out the effect of the environment on the roots before transplanting. Rice has to be transplanted, and before the transplanting period, the roots, stems and leaves of rice have been basically grown, and storage organ growth occurs after transplanting. Because the root is the first part of the plant exposed to heavy metals, the enrichment of heavy metals is stronger in the roots than in the other parts. Studies have also found that rice roots are the parts that are most sensitive to heavy metal stress, so the most sensitive parameter is WRT. However, if we use WRT alone, the effects of heavy metal stress would be overestimated. The stems and leaves are grown before transplanting, so there is no assurance that their growths are entirely affected by the post-transplant environment. However, the storage organs of rice are unique as they grow during the jointing–booting stage. Therefore, it can be concluded that their growth status has little to do with the environment before transplanting, and it is largely due to the new environment. So it is not inaccurate to use WRT or WSO alone to determine the level of heavy metal stress in rice. As shown in Figure 8, no matter what scale, WRT exaggerates heavy metal stress relative to WSO. Therefore, the SORMR index was applied to weaken the impacts of the background factors on rice. Other crops will also experience heavy metal pollution caused by the environment; therefore, SORMR can also be used to monitor heavy metal stress in other crops. In conclusion, the applicability of this finding in other areas and to other crops should be further confirmed.

We found that the optimal scale to assess heavy metal stress in rice is limited to high resolution, and it is likely that the outcomes of the different characteristics of the different monitoring objects will be different. However, this method can still be selected for other characteristics and crops. According to the results of SORMR, it can be clearly seen that 8 m and 16 m are suitable resolutions for monitoring heavy metal stress, and 16 m is the optimal scale at high resolutions. Garrigues [22] noted that farmland is a vegetation cover type with strong spatial heterogeneity, so the selected image scale should be small enough to minimize spatial heterogeneity. Marceau [57] indicated that too high of a resolution may also increase the spectral variability within the category and reduce the classification accuracy. Therefore, compared with several other scales at the Zhuzhou test site, 16 m should be the point where the spatial heterogeneity and the spectral variability are minimized. The ratio of SORMR clearly indicates that the spatial resolution should be chosen based on the characteristics of the object of interest. Huang [43] scaled up an eight meter remote sensing image to obtain 16 m, 32 m, 64 m, 128 m, 256 m images to be used for the selection of the optimal scale, but there must be a difference between the original image and the upscaled image. Choosing an upscaled image to study the optimal scale in a practical application would result in errors. Due to the relatively small area of the rice paddy fields in this study, large-scale images could not be selected to study the appropriate scale. In the future, we will continue to combine the scale problem with heavy metal stress to determine the similarities and differences between the upscaled image and original image.

In this study, SORMR was the only index used to monitor heavy metal stress, but it was not the only index used to identify the optimal scale. Through the comparison of WRT, WSO and SORMR, it was found that 16 m is the optimal scale at high resolutions. Multiple indexes can further reduce the uncertainty of the factors and improve the accuracy of the results.

## 6. Conclusions

In this study, the new SORMR index was calculated to monitor heavy metal stress based on the improved WOFOST assimilation framework. By comparing the spatial–temporal changes in SORMR under different stress levels, we found that heavy metal stress affected WRT and WSO to a large extent throughout the entire crop growth period. Therefore, SORMR can successfully be used as an index to monitor heavy metal stress. By comparing the ratios of SORMR at different resolutions, we found that the degree of heavy metal stress differed at different spatial resolutions. In addition, the results of the current study suggest that 8 m and 16 m were suitable scales for monitoring heavy metal stress in rice, and 16 m is considered the optimal scale to assess heavy metal stress in rice at high resolutions.

## Figures and Tables

**Table 1:** The heavy metal concentrations in the two study areas.

Study Area	Type	Cd	Hg	Pb	As	Pollution Level
A	Soil	0.84	0.25	78.32	10.22	Low
	Rice Tissue	0.82	0.04	10.60	5.39	
	Pollution index	0.59	1.25	0.95	0.53	
B	Soil	3.28	0.51	120.75	18.15	High
	Rice Tissue	5.90	0.06	36.73	7.04	
	Pollution index	2.29	2.55	1.46	0.95	
Background value		1.43	0.2	82.78	19.11	

Note: The background value of the heavy metal content was obtained from the Hunan Institute of Geophysical and Geochemical Exploration. The pollution index is equal to the soil heavy metal content divided by the background value. The unit of the heavy metal concentrations in the study areas is mg/kg.

**Table 2:** The attributes of the remote sensing images.

Sensor	Spatial Resolution (m)	Revisitation Period (day)	Breadth (km)	Band 1 (nm)	Band 2 (nm)	Band 3 (nm)	Band 4 (nm)
GF-1	2	4	60	0.45~0.90			
	8	4	60	0.45~0.52	0.52~0.59	0.63~0.69	0.77~0.89
	16	2	800	0.45~0.52	0.52~0.59	0.63~0.69	0.77~0.89
HJ-1	30	4	360 (1CCD) 700 (2CCD)	0.43~0.52	0.52~0.60	0.63~0.69	0.76~0.90

**Table 3:** The minimum, mean and maximum values of the stress factor f.

Statistical Analysis	A_30m	B_30m	A_16m	B_16m	A_8m	B_8m	A_2m	B_2m
Mean	0.819	0.781	0.8447	0.7484	0.775	0.691	0.7518	0.704
Minimum	0.775	0.6	0.705	0.6	0.664	0.6	0.6	0.6
Maximum	0.9171	0.95	0.95	0.8161	0.9462	0.783	0.8851	0.775

**Table 4:** The minimum, mean, maximum, and standard deviation values of the SORMR values.

Statistical Analysis	A_30m	B_30m	A_16m	B_16m	A_8m	B_8m	A_2m	B_2m
Minimum	0.00030	0.00029	0.00031	0.00028	0.00029	0.00027	0.00029	0.00027
Mean	0.73376	0.75426	1.72085	0.7826	0.76162	0.81404	0.77959	0.81175
Maximum	4.55944	4.6869	4.47896	4.86346	4.73293	5.05791	4.84456	5.04432
Standard Deviation	1.23371	1.26862	1.21164	1.31682	1.28115	1.37043	1.31164	1.3665
